# Prediction of Mechanical Properties of Artificially Weathered Wood by Color Change and Machine Learning

**DOI:** 10.3390/ma14216314

**Published:** 2021-10-22

**Authors:** Vahid Nasir, Hamidreza Fathi, Arezoo Fallah, Siavash Kazemirad, Farrokh Sassani, Petar Antov

**Affiliations:** 1 Department of Mechanical Engineering, The University of British Columbia (UBC), Vancouver, BC 2054-6250, Canada; vahid.nasir@alumni.ubc.ca (V.N.); sassani@mech.ubc.ca (F.S.); 2 School of Mechanical Engineering, Iran University of Science and Technology, Tehran 16846-13114, Iran; hrfathi93@gmail.com; 3 Department of Mechanical Wood Technology, Faculty of Forest Industry, University of Forestry, 1797 Sofia, Bulgaria

**Keywords:** wood characterization, mechanical properties, photodegradation, artificial weathering, color change, ultraviolet radiation, machine learning

## Abstract

Color parameters were used in this study to develop a machine learning model for predicting the mechanical properties of artificially weathered fir, alder, oak, and poplar wood. A CIELAB color measuring system was employed to study the color changes in wood samples. The color parameters were fed into a decision tree model for predicting the MOE and MOR values of the wood samples. The results indicated a reduction in the mechanical properties of the samples, where fir and alder were the most and least degraded wood under weathering conditions, respectively. The mechanical degradation was correlated with the color change, where the most resistant wood to color change exhibited less reduction in the mechanical properties. The predictive machine learning model estimated the MOE and MOR values with a maximum R^2^ of 0.87 and 0.88, respectively. Thus, variations in the color parameters of wood can be considered informative features linked to the mechanical properties of small-sized and clear wood. Further research could study the effectiveness of the model when analyzing large-sized timber.

## 1. Introduction

Nondestructive evaluation (NDE) of wood is crucial for monitoring purposes and timber grading [1,2], especially when the wood is used in load-carrying applications. The characterization of the mechanical properties of wood, including the modulus of elasticity (MOE) and modulus of rupture (MOR), can be performed using tensile, compression, and bending static tests [3]. However, these methods are costly and time-consuming, and are not suitable for in situ characterization and monitoring purposes. While visual strength grading is being practiced in some industrial applications [4], fast and reliable assessment of timber and wood-based materials that accounts for material variability and anisotropic properties and natural defects requires the application of NDE methods. 

Near-infrared (NIR) spectroscopy is one of the most commonly used NDE methods for wood characterization. NIR spectroscopy is sensitive to changes in the chemical composition of wood [5] and has been used for wood classification and the estimation of different wood properties [6,7,8,9,10]. Wave propagation-based methods have also been employed to estimate timber MOE [11,12]. For example, a wave is generated in wood using an impact or piezoelectric actuators, respectively, in the stress wave or ultrasonic wave methods. The propagated wave velocity, and consequently the wood dynamic MOE, are typically calculated in wave propagation techniques using the time-of-flight method. The prediction of the mechanical properties [13,14] and the detection of internal check formation [15,16] in weathered thermally modified timber have been performed using the ultrasonic wave method. The physical and mechanical properties of thermally modified wood have also been predicted using the stress wave method [17]. Recently, the elastic and viscoelastic properties of wood have been characterized using the Lamb wave propagation method [18,19]. Another readily available NDE method for wood classification and characterization is related to measuring the surface color of wood.

The surface color is affected when the wood is used in applications that cause changes to its chemical composition, such as through thermal treatment, or ultraviolet (UV) or laser irradiation [20,21]. The color change has been shown to be a quality indicator for thermally modified timber (TMT) [22,23]. It has been reported that color change can be linked to the intensity of thermal treatment [24,25,26,27,28,29] and the mechanical properties of TMT [30]. The correlation between the color change and the pressure treatment of wood has also been reported in the literature [31]. Color measurement has also been employed for the classification [32] and characterization [33,34] of thermally modified timber. The color is also an important feature to be studied during the wood weathering since the weathering of wood causes photodegradation. The CIELAB color measuring system has been widely employed to study the color change in wood under UV radiation [35,36,37,38,39,40,41,42]. The color was considered as an informative parameter to monitor the wood photodegradation [43]. Additionally, the infrared spectroscopy analysis of wood has been used to assess the change in wood chemical composition under weathering [44,45,46,47,48]. The critical role of color change during wood weathering may suggest further investigations to see if the color change can be used as a quality control tool for monitoring the in-service weathered wood. One of these monitoring tasks is to assess the feasibility of using the color change for predicting the MOE and MOR of weathered wood.

The mechanical degradation of wood under weathering depends on different factors. These factors include but are not limited to the type of wood, weathering condition (UV radiation, temperature, humidity, rain, etc.), thickness of wood under weathering, and conditioning parameters. It has been reported that UV and solar irradiation result in the degradation of the mechanical properties in thin wood strips [49,50,51]. Accelerated UV exposure can result in a 20–40% drop in the strength of wood strips [52]. The mechanical degradation in wood-based composites under weathering conditions with a panel thickness of 8 mm and 12.5 mm has also been reported in the literature [53,54]. Weathering has also been reported to cause a reduction in the impact bending properties of spruce, fir, and oak wood with a board thickness of 20 mm [55]. The impact of weathering on the mechanical properties of full-size timber has also been investigated [56]. van Blokland et al. [14] studied the impact of natural weathering on the mechanical properties of Norway spruce timber. The timbers were conditioned after the weathering and mechanically tested. They reported that the bending strength of the control and thermally treated spruce were reduced after weathering by 6% and 9%, respectively. A 4% reduction in the MOE of both types of timbers was also reported.

Apart from the size of the wood cross-section, the type of wood species, weathering situations, and conditioning also impact the mechanical properties of weathered wood. Tomak et al. [57] showed that the reduction in the mechanical properties under weathering is significantly dependent on the type of wood species. They reported that while the MOR of Ash wood decreased by 18% after 24 months of weathering, Iroko wood experienced a 40% MOR reduction in the same time period. The mechanical degradation of softwood and hardwood under weathering can also be significantly different [57]. The impact of weathering is greatly affected by the environmental conditions. In the case of artificial weathering, for example, the impact of UV radiation is affected by the moisture content (MC), relative humidity (RH), and temperature conditions. Timar et al. [36] reported that the combination of UV radiation and temperature could cause lignin degradation, while exposure of wood to temperature alone did not affect the lignin. Persze and Tolvaj [58] also reported that the photodegradation of wood is affected by temperature. Therefore, while UV radiation alone is mainly known to be a phenomenon causing surface damage, its impact on the wood can be aggravated when combined with harsh environmental conditions. To study the effect of UV radiation, the exposed samples are conditioned after the weathering test. This is useful to understand the mechanism of damage caused; however, some researchers did not condition their samples after the weathering and before mechanical testing to simulate the practical situations [56]. For example, Boonstra et al. [56] showed that, compared to the conditioned samples after weathering, the weathered samples that were directly tested without conditioning showed higher reduction in the MOE and MOR. While such an approach assesses the combined effect of different parameters (UV radiation, temperature, MC change, etc.) on the mechanical properties of wood under weathering, this is more aligned with real situations, where in situ monitoring of in-service weathered wood structures is intended.

The above discussion shows that it is hard to generalize the mechanical behavior of wood under weathering conditions, since it depends on many factors. The mechanical behavior of the weathered wood depends on the size of wood cross-section, type of wood species, and range and intensity of environmental parameters such as the temperature and conditioning situations. Therefore, finding an NDE method for monitoring the mechanical behavior of weathered wood becomes a crucial quality control task. The MOE and MOR of defect-free wood subjected to UV radiation were recently estimated using the Lamb wave propagation method [59,60]. Yet, the color parameters of wood have not been used to make an intelligent monitoring model to predict the MOE and MOR of degraded wood under weathering. The use of color parameters to monitor the mechanical properties of photodegraded wood may offer a fast, cost-effective, and reliable complement to wave propagation-based methods.

The aim of this research work was to link the color change of weathered wood to its mechanical properties and develop a machine learning-based model for monitoring the MOE and MOR of weathered wood. The weathering condition consisted of UV radiation at 40 °C. This is a very common temperature in regions with hot climate, such as Iran. The weathered samples were not conditioned after the weathering to better simulate the practical conditions. Thus, the weathering impacted the samples not only due to UV radiation, but also as a result of the temperature conditions. Since the objective of this study was to develop an NDE tool for the in situ monitoring of weathered wood and the evaluation of the combined effect of all parameters involved during the weathering on its mechanical properties, finding the UV penetration depth or separating the share of different playing factors on the mechanical degradation of wood was not within the scope of this study. 

## 2. Materials and Methods

### 2.1. Sample Preparation

**Figure 1:**
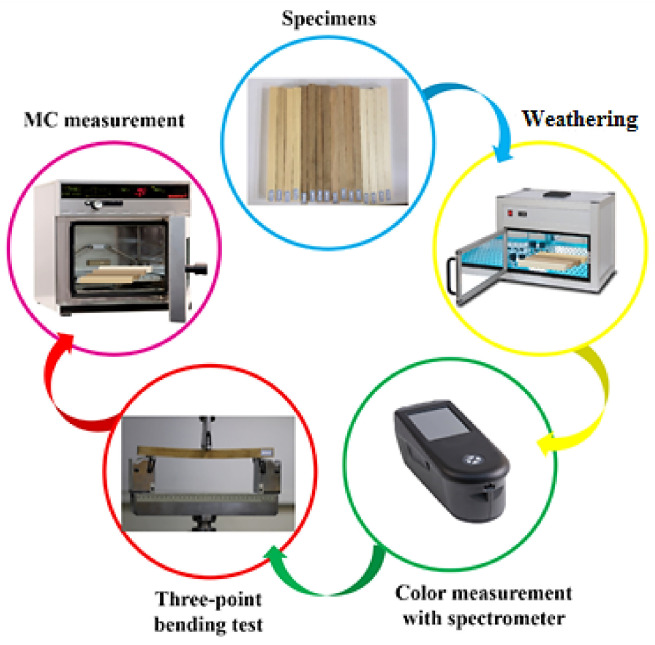
The experimental procedure employed in this study.

In this study, twenty samples of poplar (*Populus euroamerican*), alder (*Alnus glutinosa*), oak (*Quercus spp*.), and fir (*Abies alba*) were prepared, resulting in a total of eighty samples. The samples were clear, with no types of defects such as knots. The dimensions of the flat-sawn samples in the radial, tangential, and longitudinal directions were 20 mm × 20 mm × 300 mm, respectively, according to ISO-13061-3 and -4 standard methods [61,62]. The samples were divided into groups of four specimens from each wood species. One group was considered as the control and was not exposed to artificial weathering. Other groups were exposed to weathering for different time periods. Considerations were made to ensure that there was no significant variation of wood initial properties between the different groups according to the procedure explained in [60] based on guided wave propagation method. Figure 1 illustrates the experimental procedure employed in this study. 

### 2.2. Weathering Test 

In the present study, the wood samples were placed in a weathering chamber. Weathering experiments were conducted immediately after the sample preparation. Samples of each group from each wood species were exposed to UV radiation for 24, 100, 150, and 240 h, respectively. The chamber had two lamps (OSRAM HQE-40 Hg, Munich, Germany) with a length, diameter, and a spectrum of 90 mm, 10 mm, and 240–570 nm, respectively, with a radiated power in the wavelength range of 315–400 nm. The wood samples were placed at a distance of 500 mm from the UV light source. Before the exposure to UV radiation, the samples were placed in a conditioning room with a relative humidity of 65% and a temperature of 20 °C to reach the equilibrium moisture content of 12% (standard deviation = 0.33). During the experiment, the humidity and temperature inside the chamber were controlled to be 55–60% and 40 °C, respectively. One of the longitudinal-tangential (L-T) surfaces of the wood samples was subjected to the UV radiation and then the color measurements and mechanical bending tests were immediately conducted without any further conditioning. As already explained in the Introduction section, such a method can better simulate the practical conditions, especially during the in situ monitoring tasks. The weathering conditions can degrade wood through both the UV effect and the temperature condition resulting in a change in the MC. This study does not focus on the pure effect of UV radiation on the degradation of wood samples. Mechanisms of degradation under weathering, especially UV radiation, has been discussed in the literature [36,49,58,63,64,65]. Thus, instead of analyzing the role of different governing factors in wood degradation, this study aims to evaluate the feasibility of developing monitoring models for predicting the mechanical degradation of weathered wood.

### 2.3. Measurement of Color Parameters

The color of the wood samples was evaluated using the CIELAB color measuring system. The color measurement was performed on all wood samples before and after the weathering experiment. The color measurement was performed on three locations in the center and close to the ends of the samples (30 mm away). The color measurement was carried out on the degraded surface of the wood samples and performed using a spectrophotometer (Model #CM-2600d, Konica Minolta Inc., Tokyo, Japan) with a D65 illuminant, a 10° standard observer, and a sensor head of 6-mm (ASTM D2244-16 standard [66]). Once the color coordinates (*L*, *a*, and *b*) were measured before and after the weathering tests, the difference in the lightness (ΔL) and the chromatic coordinates (Δ*b* and Δ*a*) were calculated. The total color change (Δ*E*) was obtained for each wood sample using the following equation:

(1) $$\Delta E={\left[{\left(\Delta L\right)}^{2}+{\left(\Delta a\right)}^{2}+{\left(\Delta b\right)}^{2}\right]}^{1/2}$$

The color measurement was also performed on the samples that were not placed into the weathering chamber. 

### 2.4. Mechanical Properties

The three-point bending tests were performed using a STM-1 50 testing machine (Santam Engineering Desgin Co. Ltd., Tehran, Iran). The crosshead speed was set to 1 mm/min (ISO 13061-3 and -4 [61,62]). The bending tests were performed on the longitudinal-tangential surface of the samples where the degraded plane was placed under tension. The MOE and MOR of the wood samples were calculated based on the ISO 13061-3 and -4 from the mechanical bending tests. 

### 2.5. MC Measurements 

The measurement of MC and density after weathering was done based on the ISO-13061-1 and -2 standard methods [67,68] from the cookie samples (50 mm × 20 mm × 20 mm [L, T, R]) cut from the vicinity of the wood samples. The average MC of the samples was about 12% before the weathering experiment. For this purpose, separate cookies with the same size were prepared before the preparation of the final weathering samples (20 mm × 20 mm × 300 mm). The samples prepared for MC measurements were weighed (*m*_1_) and then placed in an oven at the temperature of 101 °C for 24 h. The oven-dried weights (*m*_2_) of the samples were then measured and the MC was calculated via:

(2) $$W\;(\%)=\frac{m_{1}-m_{2}}{m_{2}}\times 100$$

where *W*, *m*_2_, and *m*_1_ are the MC, the dry mass and the wet mass of the wood samples, respectively. 

### 2.6. Statistical Analysis

The data of color change were analyzed with a one-way ANOVA in Minitab 19 (AppOnFly s.r.o., Prague, Czech Republic). For each wood species, the significance between the color changes of the samples weathered at different time periods was analyzed using Tukey’s comparison test. Having a low number of replications is a limitation of such a study. However, it should be noted that the main aim of this research was to monitor the weathered wood samples through machine learning modeling, which does not necessarily depend on the requirements for statistical analysis.

### 2.7. Machine Learning Analysis

**Figure 2:**
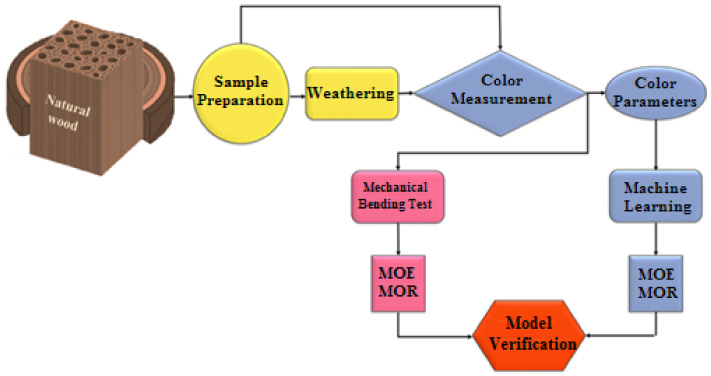
Flowchart of the proposed methodology to monitor the mechanical properties of the weathered wood by color measurement.

Figure 2 illustrates the flowchart of the adopted methodology in this study to estimate the mechanical properties of weathered wood through machine learning (ML) modeling. Different ML models and artificial neural networks (ANNs) have been employed for damage/defect detection [69,70], prediction of the material’s properties [71,72], and process condition monitoring [73,74,75,76]. Decision tree regression modeling was used in this study for the prediction of the mechanical properties of wood samples. While ANNs are challenging to interpret, the outputs of decision tree models can be comprehended easier. Furthermore, the significance of predictor variable can be identified in the model, which helps to study the relationships between the features and take care of the redundant features with relatively lower importance [77]. The current study dealt with a small dataset; however, decision tree modeling has been successfully employed in the literature to analyze small datasets [15,59,78] when predicting the mechanical properties or checks formation in weathered wood samples. The classification and regression trees (CART) algorithm [79] was used in this study. The decision tree development process included tree growing and pruning phases. In the tree growing phase, the node splitting criteria were based on the highest contribution to lowering the least squared error during the model training. The optimal tree was chosen in the pruning phase as the smallest tree that has an R^2^ within one standard error of the tree representing the highest R^2^ obtained during the model validation. The 5-fold cross-validation method was employed in this study. The details of the developed decision tree model were similar to those chosen in [59] and based on the discussion provided in [80].

The contribution of each feature (either as a primary splitters or surrogate one) in improving the performance of the model was studied based on minimizing the least squared error. The most important feature causes the highest improvement to the model and the other features are relatively ranked, accordingly. Additionally, clustering analysis was employed to observe the level of similarity and find the common characteristics between the color parameters, MOE and MOR, using agglomerative hierarchical clustering as explained by Fathi et al. [81].

## 3. Results

### 3.1. Mechanical Degradation under Artificial Weathering

**Figure 3:**
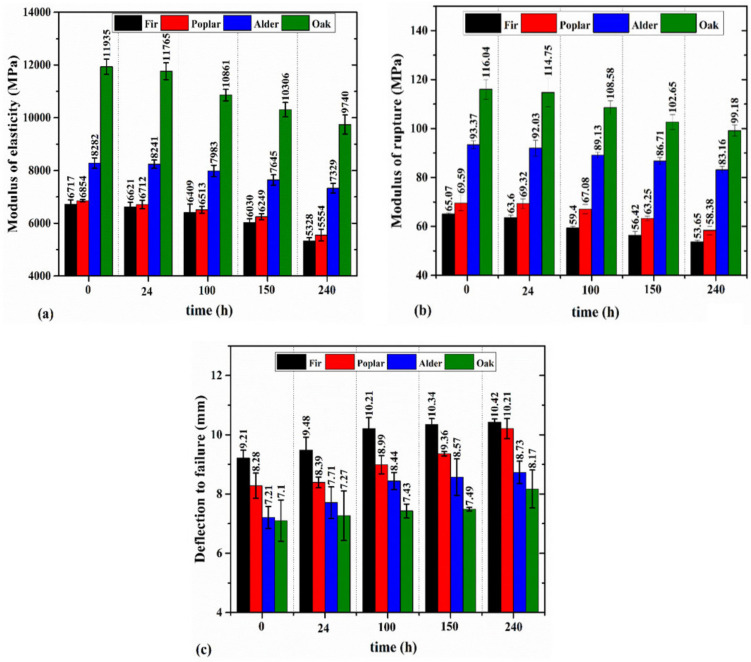
The variation of MOE (**a**), MOR (**b**), and deflection to failure (**c**) in weathered wood samples.

The MC and density of the wood samples decreased under weathering. Following 240 h of exposure, fir, alder, oak, and poplar wood experienced a 4.9%, 3.2%, 4.7%, and 8.1% reduction in the density, respectively. The initial MC of fir (12.1%), alder (11.6%), oak (12.4%), and poplar (12.1%) were also reduced to 6.3%, 5.3%, 5.8%, 5.3%, respectively, after 240 h of weathering. Ouadou et al. [49] reported that 120 h of weathering resulted in the mass loss due to the reduction of the MC. Figure 3 shows the impact of weathering on the MOE, MOR, and deflection to failure of different wood samples. It shows that the MOE and MOR decreased with increasing weathering duration. Overall, alder was shown to be the most resilient wood with an 11.5% and 11% reduction in its MOE and MOR after 240 h of weathering. Fir also exhibited the highest degradation, with a 21.5% and 17.5% reduction in its MOE and MOR after 240 h of weathering. The mechanical degradation under ultraviolet conditions was discussed to affect the microstructure of wood and its effect on the chemical composition and results in degradation of lignin [49]. Additionally, Persze and Tolvaj [58] explained that the higher temperatures increase the wood degradation under ultraviolet conditions. The mass loss of wood may also occur during the weathering [49]. Boonstra et al. [56] reported that wood specimens conditioned after weathering showed lower reduction in the mechanical properties. Thus, the mechanical degradation observed in Figure 3 is affected by the combined impact of the UV radiation and temperature condition resulting in mass loss due to reduction in the MC.

**Figure 4:**
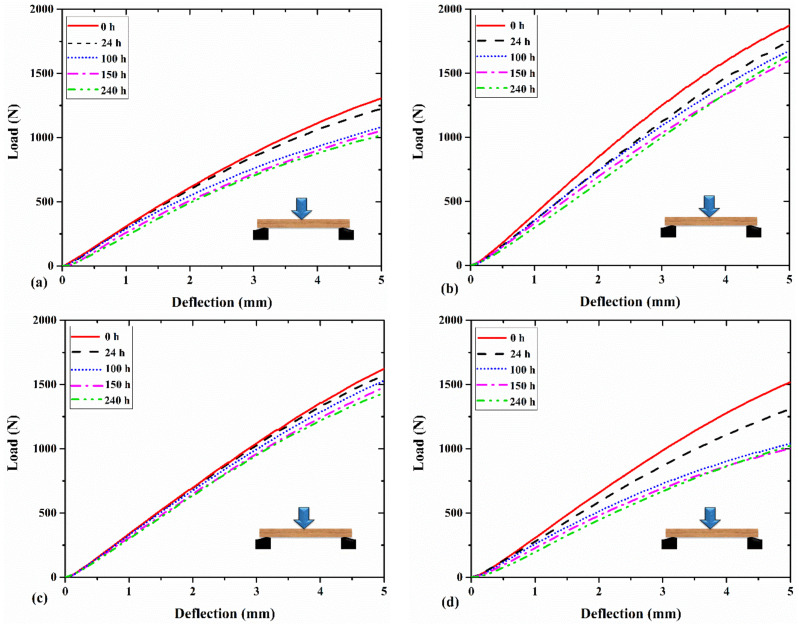
The load–deflection diagram for (**a**) fir, (**b**) alder, (**c**) oak, and (**d**) poplar wood samples exposed to different times of weathering.

**Figure 5:**
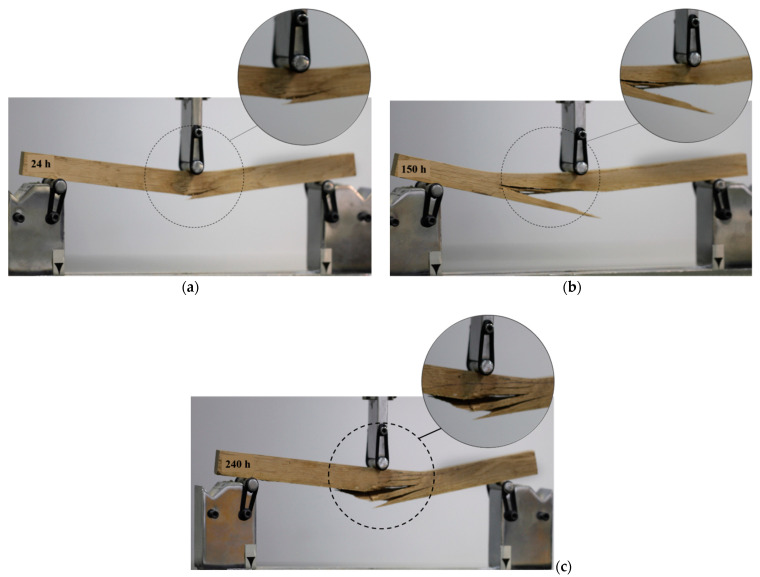
The fracture plane of the weathered oak wood at three exposure times: 24 h (**a**), 150 h (**b**), and 240 h (**c**) under the static bending test.

Despite the reduction in the MOE and MOR, weathering led to an increase in the deflection to failure in all wood species. This increment was 13%, 23%, 21%, and 15% for fir, poplar, alder, and oak wood after 240 h of exposure. This observation may be linked to the impact of ultraviolet condition on enhancing the viscoelasticity of wood. Fathi et al. [60] showed that the loss modulus and loss factor of the wood samples tested in this study increased under ultraviolet condition, proving that wood exhibits more viscoelasticity when exposed to UV degradation. This may result in an increase in the deflection to failure in the degraded samples. Feist [64] reported the leaching and plasticizing effects of water during the weathering process. This plasticizing effect can also facilitate the enlargement of micro-checks. Figure 4 shows the load-deflection diagram of the wood samples after different weathering time periods. It shows that the degradation resulted in having a larger deflection at certain load levels. It was also indicated that after 150 h or 240 h of exposure, the fir and poplar wood samples reached a deflection of 5 mm at a load level of around 1000 N. However, this deflection was achieved at a higher load level (~1500 N–1600 N) for hardwoods such as alder and oak. Apart from its impact on the MOE, MOR, and deflection to failure, the degradation affected the failure modes. Figure 5 shows the failure modes after the static bending tests for the oak samples under weathering at three exposure time periods. It was observed that the failure mode was mainly governed by the tensile failure perpendicular to the grain in the radial-longitudinal plane (fiber compression was also observed in parallel). Consequently, increasing the exposure time resulted in having a deeper brittle fracture and splinter in tension. This can be due to the destruction of the middle lamella [65], which contains significant lignin content. Severe checking may develop with longer exposure times in the cell wall components resulting in the loosening and detaching of the fibrils and tracheids from the surface [65]. Feist [64] discussed that the weathering condition can result in the destruction of the middle lamella and cell wall layers. This can impact the cohesive strength of wood tissue [65] that may result in the mechanical degradation of the wood as well. 

### 3.2. Color Change 

**Figure 6:**
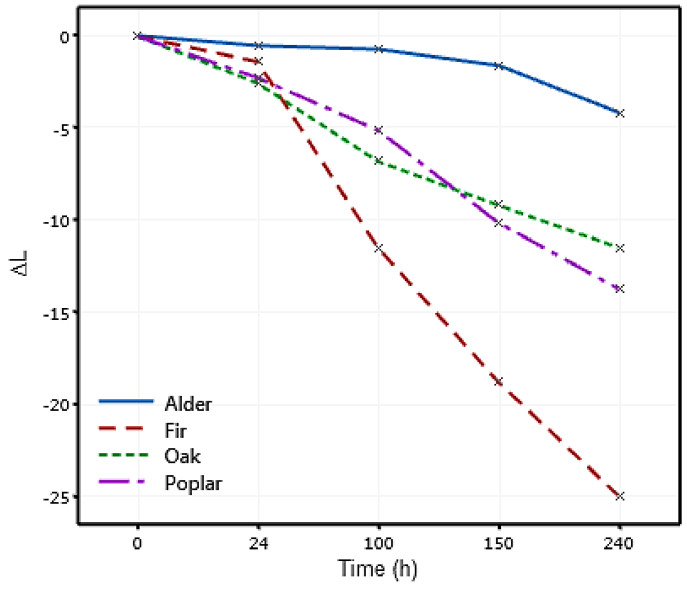
Variation in the lightness (Δ*L*) of different wood species under artificial weathering.

Tolvaj and Faix [82] stated that the light reflection decreased after UV radiation, resulting in the wood seeming darker. The reduction in the lightness of the samples with the exposure time can be linked to the negative Δ*L*, as shown in Figure 6. A similar trend of darkening of the wood appearance after UV radiation has been reported in the literature [35,82]. It can be seen in Figure 6 that after 24 h of exposure, there was a trivial change in the lightness with a minor difference between different wood species. In this time, poplar and oak showed more darkening (Δ*L* = −2.5). By increasing the exposure time, fir underwent a significant change in Δ*L* while alder showed a minimal change in Δ*L*. After 240 h, alder accounted for the minimum change in the lightness (Δ*L* = −4) followed by oak (Δ*L* = −11.5) and poplar (ΔL = −13.7), and the maximum change in the lightness occurred in fir wood (Δ*L* = −25). 

**Figure 7:**
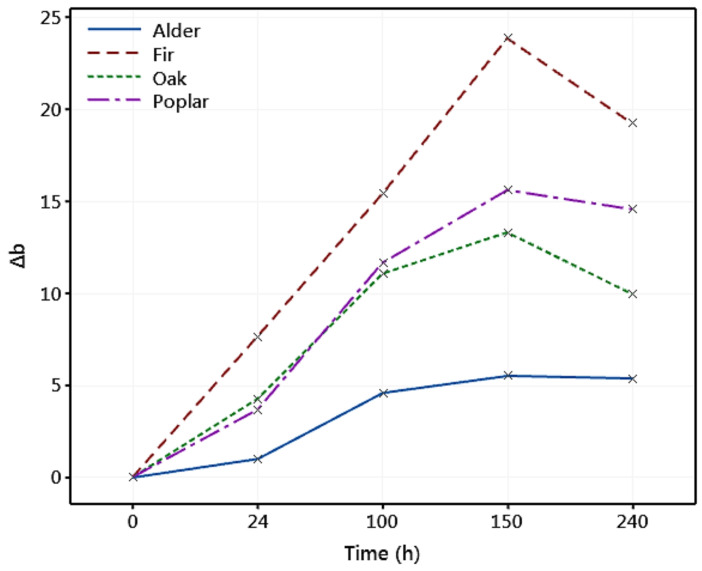
Variation in the yellowness (Δ*b*) of different wood species exposed to artificial weathering.

Variation in the yellowness (Δ*b*) of the wood samples exposed to weathering is shown in Figure 7. It can be seen that the yellowness increased with exposure time and reached its maximum value after 150 h. After this time, fir poplar, and oak experienced a slight reduction in the yellowness, while it stayed almost unchanged for alder. The increase in the yellowness of the samples was discussed to be mainly due to the degradation of lignin [35,36,46]. The continuous increase of the yellowness followed by a slight reduction under artificial weathering was reported by Timar et al. [36] and Pandey [83]. Figure 7 indicates that lignin degradation occurred even after 24 h of exposure. The fir wood accounted for the highest rate of increment in the yellowness following the exposure, with the highest Δ*b* occurring after 150 h of exposure (Δ*b* = 23.8). On the other hand, the smallest change in the yellowness was observed in alder, suggesting a lower lignin degradation of alder wood under ultraviolet conditions compared to other species. The variation in the yellowness was almost similar in the first 100 h of the exposure for poplar and oak wood. For longer exposure times (i.e., 150 h and 240 h), the yellowness of poplar was relatively higher than that for oak.

**Figure 8:**
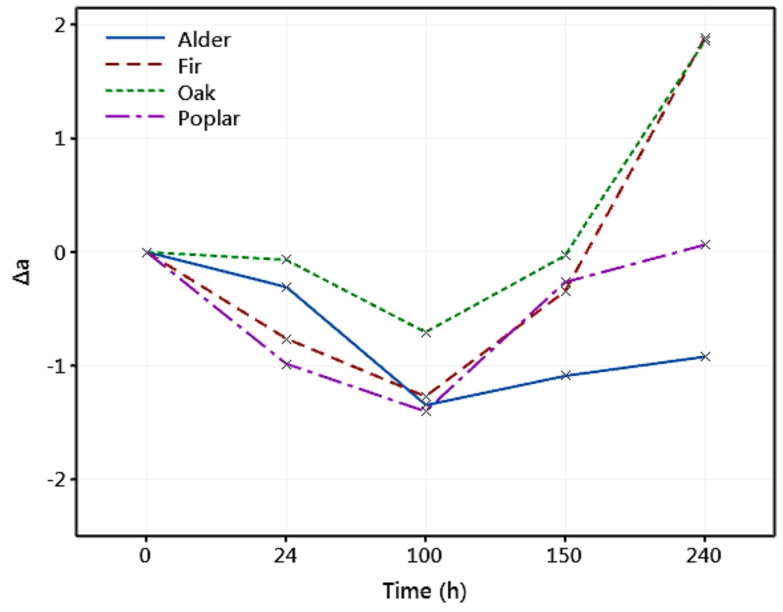
Variation in the redness (Δ*a*) of different wood species exposed to artificial weathering.

The variation in the redness (Δ*a*) of the degraded wood samples is shown in Figure 8. It can be seen that there was a reduction in the redness of all wood species during the first 100 h of exposure. However, there was an increasing trend in the redness of the samples after 100 h. While Tolvaj and Faix [82] discussed that it is challenging to explain the wood color change in a definite way, a correlation between the Δ*a* and the extractives content in wood has been reported in the literature [41,84]. For example, Persze and Tolvaj [58] showed that the extractives content has an important role in thermal decomposition during photodegradation. They also reported that the Δ*a* is higher at elevated temperatures, whereas the thermal effect does not alter yellowing. It is also indicated in Figure 8 that after 240 h, fir and oak accounted for the highest increase in the redness (Δ*a* = 1.9), while alder experienced a reduction in it. The color change parameters and the total color change for all wood species (Δ*E*) are listed in Table 1. It was observed that the total color change increased with the exposure time. A general trend for Δ*E* of fir > poplar > oak > alder was observed at all exposure times. Interestingly, fir that experienced the maximum color change showed the highest reduction in the mechanical properties, and the alder with the smallest total color change showed the minimum mechanical degradation. This indicates that the color change may be correlated with the mechanical degradation of wood and be used to predict MOE and MOR. 

### 3.3. MOE and MOR Prediction 

**Figure 9:**
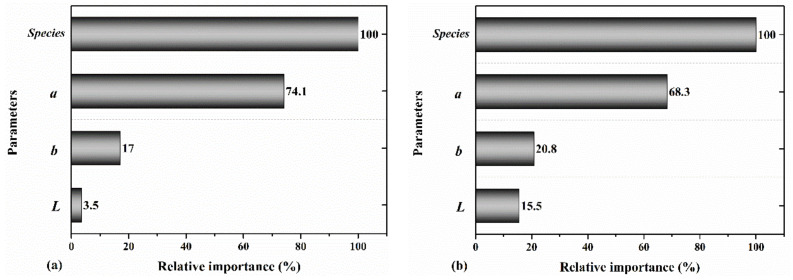
The relative importance of the wood species, *a, b*, and *L* for predicting the (**a**) MOE and (**b**) MOR.

The wood color parameters and type of the wood species were used as the input of the decision tree model to predict MOE and MOR. The results of machine learning modeling are shown in Table 2. It was observed that when the wood species type was combined with *a*, *b*, and *L*, the developed decision tree model predicted the mechanical properties of wood with an R^2^ of 0.84 and 0.77, respectively (test data). Figure 9 illustrates the relative importance of the selected input parameters used in the decision tree model and indicates that all of them were significant in developing the predictive model. It was shown that following the type of wood species, the level of redness and yellowness had the highest relative importance.

**Figure 10:**
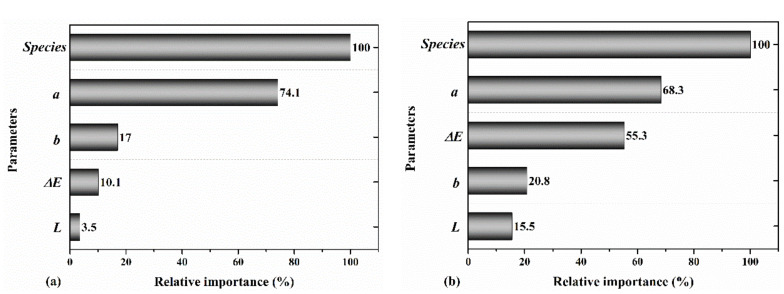
The relative importance of wood species, *a*, *b*, *L*, and Δ*E* for predicting the (**a**) MOE and (**b**) MOR.

Table 2 shows that adding the total color change Δ*(E)* parameter as an input to the model improved the prediction accuracy. In this case, the MOE and MOR were predicted with an R^2^ of 0.87 and 0.88, respectively (test data). Figure 10 shows the relative importance of the parameters used in this model. It was indicated that all of the input parameters were important in the performance of the model, while the total color change had relatively a higher importance for the prediction of the MOR than MOE. It was also observed in Figure 9 and Figure 10 that the lightness parameter had higher relative importance when predicting the MOR than MOE.

**Figure 11:**
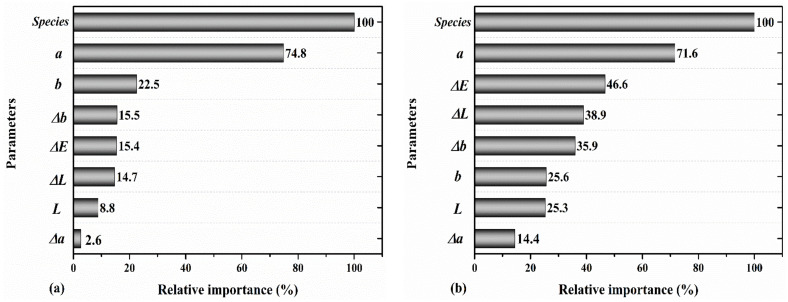
The relative importance of wood species, *a*, *b*, *L*, Δ*E*, Δ*a*, Δ*b*, and Δ*L* for predicting the (**a**) MOE and (**b**) MOR.

The last model, which used the wood species, color parameters (*a*, *b*, *L*), total color change (Δ*E*), and variation in the color parameters Δ*(a, Δb*, and Δ*L*), did not make a noticeable improvement in the prediction accuracy of MOE (R^2^ = 0.88 for the test data) and MOR (R^2^ = 0.90 for the test data). Overall, adding the three parameters Δ*a, Δb*, and Δ*L* had a minor positive impact in the performance of the model. Figure 10 and Figure 11 indicate that the wood species type and redness were still the most important parameters for the prediction of the MOE and MOR. However, in this model, Δ*b* had higher importance than Δa. Markedly, while the level of redness (a), described in the literature to be linked to the extractives content, had relatively a high importance in the performance of the model, its variation (Δ*a*) exhibited less importance in the performance of the predictive model. Overall, these findings indicate that all of the selected input parameters contributed positively to the predictive accuracy of the ML model, albeit with different levels of relative importance. These findings may suggest that there could be a dependency between the mechanical properties of weathered wood and its color parameters. However, further studies with a larger sample size could better clarify the details of these dependencies, especially when combined with the chemical composition analysis.

**Figure 12:**
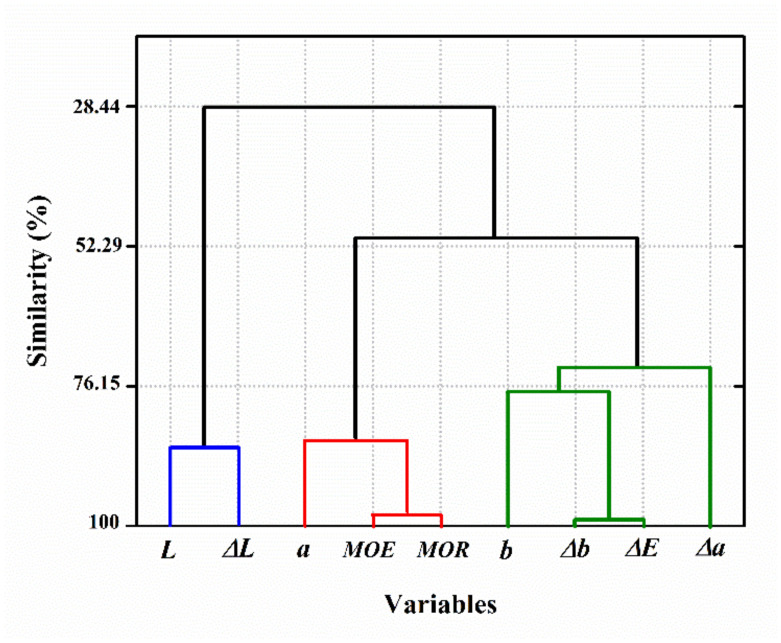
Variable clustering analysis on the color parameters, MOE and MOR (the blue, red and green lines represent the components of the CIELAB three-dimensional color space).

The results of variable clustering analysis were in accordance with those of the decision tree modeling. Figure 12 shows that the MOE and MOR had the highest similarity level with the redness, and form a cluster together. This cluster has the highest similarity with a cluster encompassing the total color change (Δ*E*), yellowness (*b*), and variation in the redness (Δ*a*) and yellowness (Δ*b*). The MOE and MOR had the lowest similarity with the lightness (*L*) and variation in it (Δ*L*), with a 28.44% similarity level. The general observations of the variable clustering analysis prove that the selected input parameters were correlated with the mechanical properties of degraded wood, which can be used for MOE and MOR prediction. Further study can be performed to outline these dependencies and similarities between the parameters when weathering conditions are changed.

## 4. Discussion and Remarks

The general trend of the mechanical degradation of the wood samples indicated the better resistance of alder and the higher vulnerability of fir to weathering condition. While it is not within the scope of a monitoring task, further research may be conducted to include the change in the chemical composition of wood to explain the role of extractives and degradation of lignin and how they can be linked to the mechanical properties of wood. The machine learning analysis showed the high importance of change in redness that is linked to the change in the extractives content in the literature. Future research considering the chemical composition of wood could better explain these observed trends. More research could also be performed to assess the relationship between the wood viscoelasticity behavior under artificial weathering and its failure mode and fracture behavior when subjected to loading. This study did not focus on evaluating the UV penetration depth, and aimed only at developing a monitoring model that reflects the combined effects of different parameters accountable for the mechanical degradation of wood under weathering. Future research aiming to explain the mechanism of degradation, rather than in situ monitoring, could be performed to separate the role of ultraviolet radiation, temperature condition, and MC and mass loss on the mechanical behavior of weathered wood. 

The performance was obtained on the basis of analyzing a small set of defect-free wood samples. The scope of the experiment could be further expanded while performing the proposed methodology on real-sized timber, including defects such as knots. One of the main challenges associated with color measurement for the characterization of wood properties is non-homogeneous surface color variation [33], especially in real-sized timber, which necessitates multiple color measurements from different locations of the timber. This is an important challenge associated with using color parameters for monitoring the mechanical properties of timber. However, due to the simplicity of the color measurement, the color features can be combined with the data acquisition from other NDE methods (such as Lamb wave propagation) to result in a robust monitoring model. Nasir et al. [59] obtained a similar range for R^2^ while using Lamb wave features for predicting the mechanical properties of weathered wood. However, their model did not need to have the type of wood species as an input parameter, as the model was able to understand the difference between the wood species through the extracted Lamb wave features. Thus, if the type of wood species to be monitored is not available, a machine learning model based on the wave propagation features may be chosen. Both the color measurement and wave propagation method should be applied to real-sized timber to have a more reliable comparative study. It should be mentioned that in real applications, other factors related to the environment (rain, decay, etc.) or the timber (grain orientation, defects, annual ring width, etc.) may impact the mechanical properties of degraded wood, and having a single monitoring method may not address both the surface and internal damages to the timber. In this case, combining color parameters that reflect the surface degradation with those of wave propagation that are linked to the internal structure of the wood can better show the mechanical degradation of large-size timber. Additionally, timber monitoring requires dataset of a larger size and perhaps other types of data-driven modeling. The choice of data-driven method depends on both the size and complexity of the data [85,86]. Different machine learning or deep learning models could be studied to choose the one that better fits the dataset of a larger size [87].

## 5. Conclusions

In this research, a machine learning model was developed based on the color parameters for predicting the MOE and MOR values of weathered wood. Artificial weathering led to a reduction in the wood mechanical properties. Wood species that experienced greater color changes exhibited a more highlighted mechanical degradation. This indicates that the color parameters may be linked to the MOE and MOR of weathered wood. Fir was shown to be more susceptible to artificial weathering, while alder was more resilient and experienced less reduction in its mechanical properties. The mechanical properties of the samples could be predicted by the color parameters, although the developed machine learning model needed the type of wood species as an input parameter for accurate prediction. It was also observed that the deflection to failure of the wood samples increased with the weathering and the failure mode under bending loading also changed. This may be due to the increased viscoelasticity of weathered wood samples. Future studies should be aimed at investigating the relation between the mechanical properties and the color parameters on large-sized timber to evaluate the reliability of the developed model based on the color parameters for NDE of timber structures. Furthermore, the chemical changes caused by weathering and the correlation between the color, mechanical and chemical properties of wood should be studied in future research. This study was performed on small and defect-free wood samples. Additionally, the sample size was small, which is a limitation of the statistical analysis. However, the decision tree modeling was able to successfully handle the dataset. Focusing on large-sized timber containing defects may require expanding the size of dataset in future studies. 

## Figures and Tables

**Table 1:** The mean value and standard deviation of the color change of wood species in different weathering time periods.

Wood	Time (hrs)	Δ*L*		Δ*a*		Δ*b*		Δ*E*	
		Mean	Std.	Mean	Std.	Mean	Std.	Mean	Std.
Fir	24	–1.39 ^a^	0.18	–0.76 ^a^	0.13	7.66 ^a^	0.63	7.82 ^a^	0.66
	100	–11.51 ^b^	1.83	–1.27 ^b^	0.18	15.42 ^b^	1.17	19.35 ^b^	0.95
	150	–18.80 ^c^	2.74	–0.35 ^c^	0.05	23.88 ^c^	1.79	30.42 ^c^	2.98
	240	–25.01 ^d^	2.58	1.89 ^d^	0.14	19.26 ^d^	1.49	31.69 ^c^	1.72
Alder	24	–0.55 ^a^	0.12	–0.31 ^a^	0.05	1.01 ^a^	0.17	1.19 ^a^	0.17
	100	–0.75 ^a^	0.18	–1.35 ^b^	0.04	4.60 ^b^	1.00	4.86 ^b^	0.92
	150	–1.63 ^b^	0.22	–1.09 ^c^	0.07	5.52 ^b^	1.46	5.89 ^bc^	1.32
	240	–4.19 ^c^	0.34	–0.92 ^c^	0.09	5.39 ^b^	1.10	6.92 ^c^	0.84
Oak	24	–2.55 ^a^	0.39	–0.07 ^a^	0.02	4.26 ^a^	1.30	5.02 ^a^	1.07
	100	–6.81 ^b^	1.06	–0.71 ^b^	0.11	11.07 ^b^	1.69	13.06 ^b^	1.50
	150	–9.20 ^bc^	1.60	–0.03 ^a^	0.01	13.32 ^b^	2.16	16.22 ^b^	2.43
	240	–11.50 ^c^	1.91	1.86 ^c^	0.37	9.97 ^b^	1.90	15.44 ^b^	1.64
Poplar	24	–2.26 ^a^	0.39	–0.99 ^a^	0.15	3.67 ^a^	0.51	4.45 ^a^	0.31
	100	–5.11 ^a^	0.94	–1.41 ^b^	0.19	11.66 ^b^	0.98	12.81 ^b^	1.26
	150	–10.14 ^b^	1.86	–0.27 ^c^	0.04	15.62 ^c^	1.42	18.72 ^c^	0.78
	240	–13.75 ^c^	2.11	0.06 ^d^	0.01	14.58 ^c^	0.94	20.12 ^c^	0.95

* Different letters (^a–d^) within a column for each wood species show the significant difference by Tukey’s comparison test (*p* < 0.05).

**Table 2:** The coefficient of determination obtained for the prediction of the wood mechanical properties using different input features.

Model Inputs	R^2^			
	MOE		MOR	
	Train	Test	Train	Test
Wood species, *a*, *b*, *L*	0.92	0.84	0.81	0.77
Wood species, *a*, *b*, *L*, Δ*E*	0.92	0.87	0.93	0.88
Wood species, *a*, *b*, *L*, Δ*E*, Δ*a*, Δ*b*, Δ*L*	0.90	0.88	0.92	0.90

## Data Availability

Not applicable.
